# Determining the critical recruitment needs for the declining population of *Olea europaea* subsp. *africana* (Mill.) P.S. Green in Free State, South Africa

**DOI:** 10.1002/ece3.10177

**Published:** 2023-06-13

**Authors:** Loyd R. Vukeya, Thabiso M. Mokotjomela, Leslie W. Powrie, L. Nenungwi

**Affiliations:** ^1^ South African National Biodiversity Institute Free State National Botanical Garden Bloemfontein South Africa; ^2^ Directorate on Biodiversity Evidence South African National Biodiversity Institute, Free State National Botanical Garden Bloemfontein South Africa; ^3^ School of Life Sciences University of KwaZulu‐Natal Pietermaritzburg South Africa; ^4^ Centre for Geographical Analysis Stellenbosch University Stellenbosch South Africa

**Keywords:** biodiversity conservation, grassland biome, habitat fragmentation, restoration, seed germination

## Abstract

*Olea europaea* subsp*. africana* (Mill.) P.S. Green (medium‐sized tree species known as “African wild olive”), provides important ecological goods and services for sustaining frugivores in the grassland biome in South Africa. We speculate that *O. europaea* subsp. *africana's* population has been declining due to habitat loss and exploitation for domestic benefits suggesting an unrecognized conservation threat. Therefore, the study aimed to investigate the anthropogenic conservation threats for *O. europaea* subsp*. africana* in the Free State, South Africa and to determine the potential importance of seed dispersal effectiveness in the restoration of the species in the study area. Overall, the results showed that 39% of the natural habitat range has been transformed by human‐mediated activities. Agricultural activities accounted for 27%, while mining activities and human settlement accounted for 12%, of natural habitat loss. In support of the study predictions, seeds of *O. europaea* subsp*. africana* had significantly higher germination and germinated faster after passing through the mammal gut (i.e., 28% and 1.49 per week), compared to other seed treatments (i.e., over 39 weeks). However, there were no statistically significant differences between seed germination of the bird‐ingested seeds, with intact fruits as the experimental control, although both were significantly greater than the de‐pulped seeds. Potential seed dispersal distances by birds were relatively larger, ranging from 9.4 km to 53 km, than those of mammals (1.5 km–4.5 km). We propose that the *O. europaea* subsp*. africana*'s habitat range may have been declining, and since it is a keystone plant species, we recommend that the complementary seed dispersal services by birds and mammals could be important for its recruitment and restoration in the degraded habitat.

## Graphical abstract

Habitat fragmentation threatens the population of Africa wild olive. Vertebrates disperse and improve seed germination restoration efforts may require the collection of seeds ingested by vertebrates to propagate seedlings.

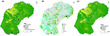

## 1 INTRODUCTION

The whole‐habitat conservation strategy has seen increasing attention in the face of global change as opposed to the traditional species‐based approaches (New, 2017; Panitsa et al., 2011; Watson et al., 2019). Pertinent to this approach, is the understanding of species' mutual interactions and their level of dependencies since it helps to identify and protect the keystone species in different environmental settings (Bond, 1994; Bronstein et al., 2004; Winfree et al., 2015). For example, the South African Grassland biome is interspersed by small to medium size fruiting woody species that support various animal species providing different ecosystem services for long time of the year (Mack & Wright, 2005; Mucina & Rutherford, 2006). These woody species support ecological succession in the grassland biome by enhancing survivorship, by providing nurse effects, and maintaining soil health through soil moisture and carbon recycling (Callaway, 1995). Healthy vegetation cover is known to improve soil properties such as bulk density, aggregate stability, and water and nutrient retention capacities, thus inherently enhancing the overall fertility of the degraded areas (Mensah, 2015; Sheoran et al., 2010).

Grasslands are threatened by fragmentation, which involves the splitting of natural habitats into smaller isolated patches primarily by human disturbances such as land clearing for human settlement (Bredenkamp et al., 2006; Carbutt & Kirkman, 2022; Matsika, 2008; Neke & du Plessis, 2004; Skowno et al., 2019; Vukeya et al., 2023), agricultural activities (Matsika, 2008; Nkuekam et al., 2018; Turpie et al., 2007), and mining activities (Brown & du Preez, 2014; Olivier, 2020). Several studies have reported that 40% of the grassland biome has been irrevocably modified while 60% of remaining grassland areas are threatened as they are losing an important aspect of their composition, structure, and function (Little et al., 2015; Neke & du Plessis, 2004; SANBI, 2013; Skowno et al., 2019), which may need urgent restoration for biodiversity conservation. In South Africa, the continuous habitat fragmentation is likely to increase biodiversity loss (Mullu, 2016), particularly for the critically endangered grassland biome species (Brown & du Preez, 2014; Matsika, 2008; Turpie et al., 2007; Vukeya et al., 2021); for example, this is observed in the southern Drakensberg Grassland (Turpie et al., 2007), and Bloemfontein Karroid Shrubland conserved in the Free State National Botanical Garden (Vukeya et al., 2023).

Several studies have highlighted the critical importance of the role of seed dispersal as a mechanism to overcome the impact of habitat fragmentation, and for the reunion of the decoupled mutualists under conservation threats (see, Buckley et al., 2006; Ibáñez et al., 2006; Jordano, 2007; Levey et al., 2005; Schupp et al., 2010). The dispersal of seeds away from their parent plants allows seedlings to colonize new sites, near or far from the parent plant, and at the same time increase the probability of surviving and becoming established adult plants in the local environment (Mokotjomela et al., 2013; Nogales et al., 2005; Schupp, 1993). The critical role of animal seed dispersal in the fast‐tracking restoration of native forest vegetation in degraded tropical lands has been reported in previous studies (Holl et al., 2017; Wunderle, 1997). Since habitat fragmentation isolates the plant populations (Haddad et al., 2015), and Rosenberg et al. (1997) indicated that effective dispersal through the habitat corridors can help to increase population persistence by allowing the continued exchange of genetic material between the connected populations.

Seed dispersal may be important to restore fragmented habitats, but not all seed dispersal is equal, and it is important for us to know how different groups contribute. Among the groups, birds and mammals are known to provide an important seed dispersal ecological service for plants (Jordano, 2000; Whelan et al., 2008; Wyman & Kelly, 2017). Vertebrates allow the ingested seeds to pass through the gut (Traveset et al., 2007; Vukeya et al., 2021), and deposit the seeds over long distances to help secure the safe microsites (Mokotjomela et al., 2013; Nathan et al., 2003; Schurr et al., 2009; Vukeya et al., 2022). However, Traveset et al. (2001) found that seeds of the Mediterranean species *Myrtus communis* appeared to significantly increase germinability after passing through the guts of birds rather than mammal species because of different seed retention times in the gut. Nevertheless, Mokotjomela et al. (2015) have demonstrated that seed dispersal effectiveness is largely determined by interactions between plant species and frugivores in a specific context. Traveset (1998) reported that large body‐size frugivores (i.e., mammals) in certain woody species negatively affect seed germination because a lengthy gut passage time in the digestive tract results in damage to seed embryos and reduced seed viability (Howe, 1986).

The fleshy fruits of *O. europaea* subsp*. africana* provides sustenance for many frugivore dispersal vectors particularly birds and mammals in South Africa (Mokotjomela et al., 2013; Sanders et al., 1997; Van Wyk & van Wyk, 2013; Vukeya et al., 2022), thus suggesting a potential to be a keystone mutualist species in terms of provision of food resources to birds (Mack & Wright, 2005; Mokotjomela, 2012; Peres, 2000). This premise was further confirmed by Vukeya et al. (2022) wherein many bird species overwintered in the patches of *O. europaea* subsp*. africana* in the grassland biome. The consumption and subsequent seed dispersal of *O. europaea* subsp*. africana* is attributed to its longest fruiting period (i.e., 10 months—grassland biome; Vukeya et al., 2022) with the large fruit crop that rewards high nutritional value entailing the proteins, lipids, and monosaccharide (Howe, 1986; Gaynor, 1994; Mokotjomela et al., 2013; Sanders et al., 1997; Vukeya et al., 2022).

It has been shown that the net seed dispersal effectiveness should be a composite value of quality components since it contributes to plant recruitment (Mokotjomela et al., 2016). However, the contributions of different key dispersal vectors are not known—such knowledge could be important in the prioritization of the conservation of different components of the habitat. The effectiveness of several dispersal agents in plant recruitment remains largely under‐explored due to the complexity of their measurement. Schupp and Fuentes (1995) indicated that most vertebrate vectors that disperse seeds might play a critical ecological role in the maintenance of plant populations. Along with the observed human‐mediated habitat fragmentation threats in the grassland biome (Matsika, 2008; Skowno et al., 2019; Vukeya et al., 2023), the quality of dispersed seeds for particular threatened plant species is ecologically important because it allows the identification of the legitimate dispersal agents. In such research, it is essential to simulate different seed dispersal mutualism networks for the sustainability and development of biodiversity conservation's adaptive management strategies.

The aim of the study was to investigate the conservation threats for *O. europaea* subsp*. africana* in Free State, South Africa and to determine whether seed dispersal may be important in the restoration of the species in the study area. The objectives of the study were: (1) To map the habitat fragmentation focusing on anthropogenic factors transforming the natural habitat for *O. europaea* subsp*. africana*; (2) To investigate the role of vertebrates (i.e., birds and mammals) for possible plant recruitment for the fleshy‐fruited tree *O. europaea* subsp*. africana*. We predicted that the seeds of *O. europaea* subsp. *africana* will have significantly higher and improved rates and speed of germination after passing through the mammal guts than seeds passing through the guts of birds because of seeds being retained for a longer time, allowing prolonged and greater scarification (Traveset et al., 2007), while the hard seed coat could also minimize the damage of the seed embryo. (3) To determine the seed dispersal distances by birds and mammals using allometric equations.

## 2 MATERIALS AND METHODS

### 2.1 Study site

**FIGURE 1:**
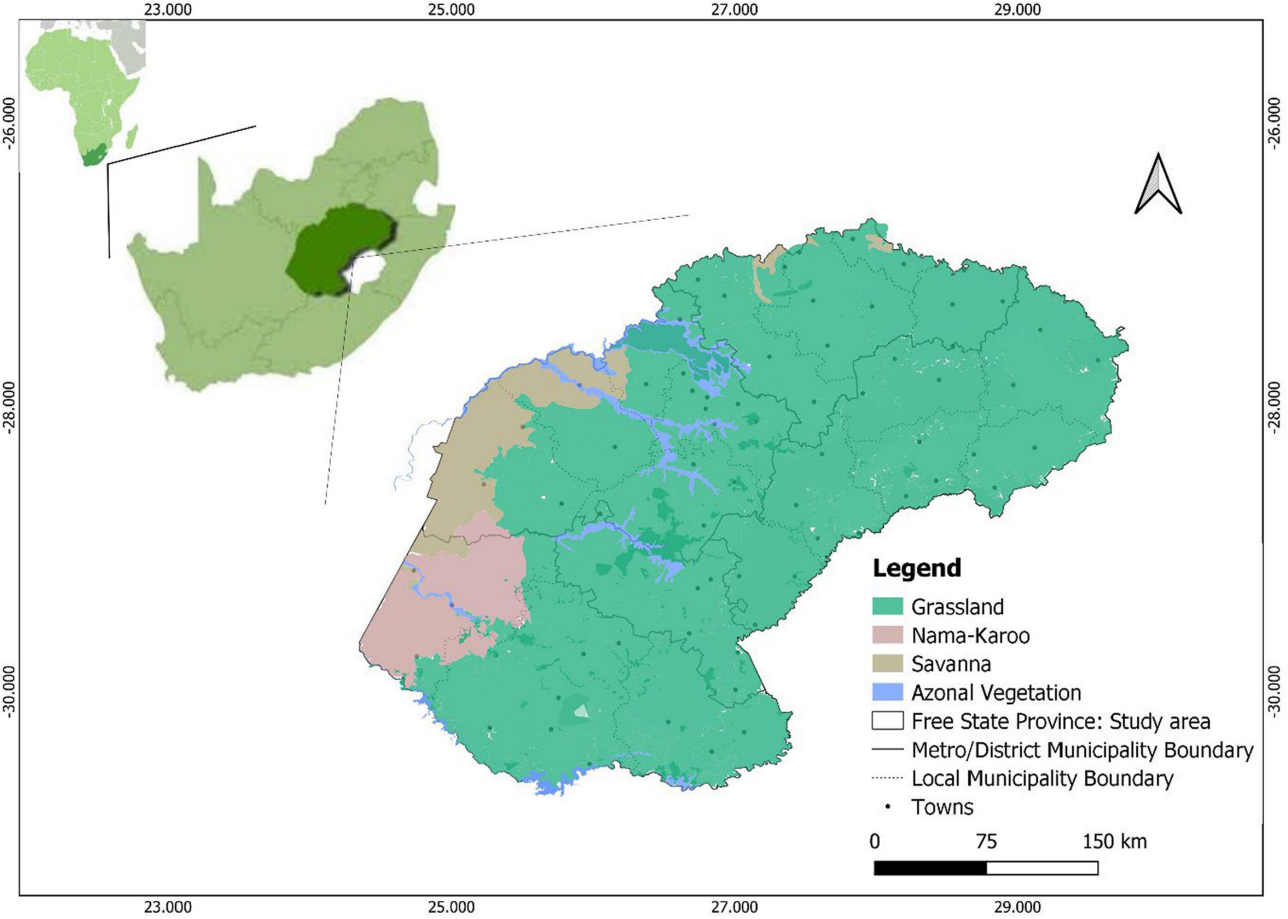
Location of the study area: Free State Province in South Africa, showing different vegetation types following Skowno et al. (2019).

The study was conducted in the Free State Province, South Africa (Figure 1). Free State is in the central plateau of South Africa (Mucina & Rutherford, 2006) and is the third‐largest province comprising 10.6% of South Africa's land area (Davis et al., 2006). The vegetation of Free State Province is characterized predominantly by the Grassland biome with a small portion of Savanna and Nama‐karoo biomes (Figure 1; Mucina & Rutherford, 2006). Free State Province land use is largely dominated by agricultural production activities, specifically crop production and domestic livestock grazing, and extensive mining and human settlement (Retief and Meyer, 2017). The province experiences a continental climate, characterized by hot summers of up to 35°C and extremely cold winters down to −8°C with frequent snowfalls and severe frost (Retief and Meyer, 2017). The province lies in the summer rainfall region of South Africa and usually receives more rain between December and March, with an average annual rainfall range between 300 and 900 mm (Moeletsi, 2010).

### 2.2 Study species: *Olea europaea* subsp*. africana* (Mill.) P.S. Green

**FIGURE 2:**
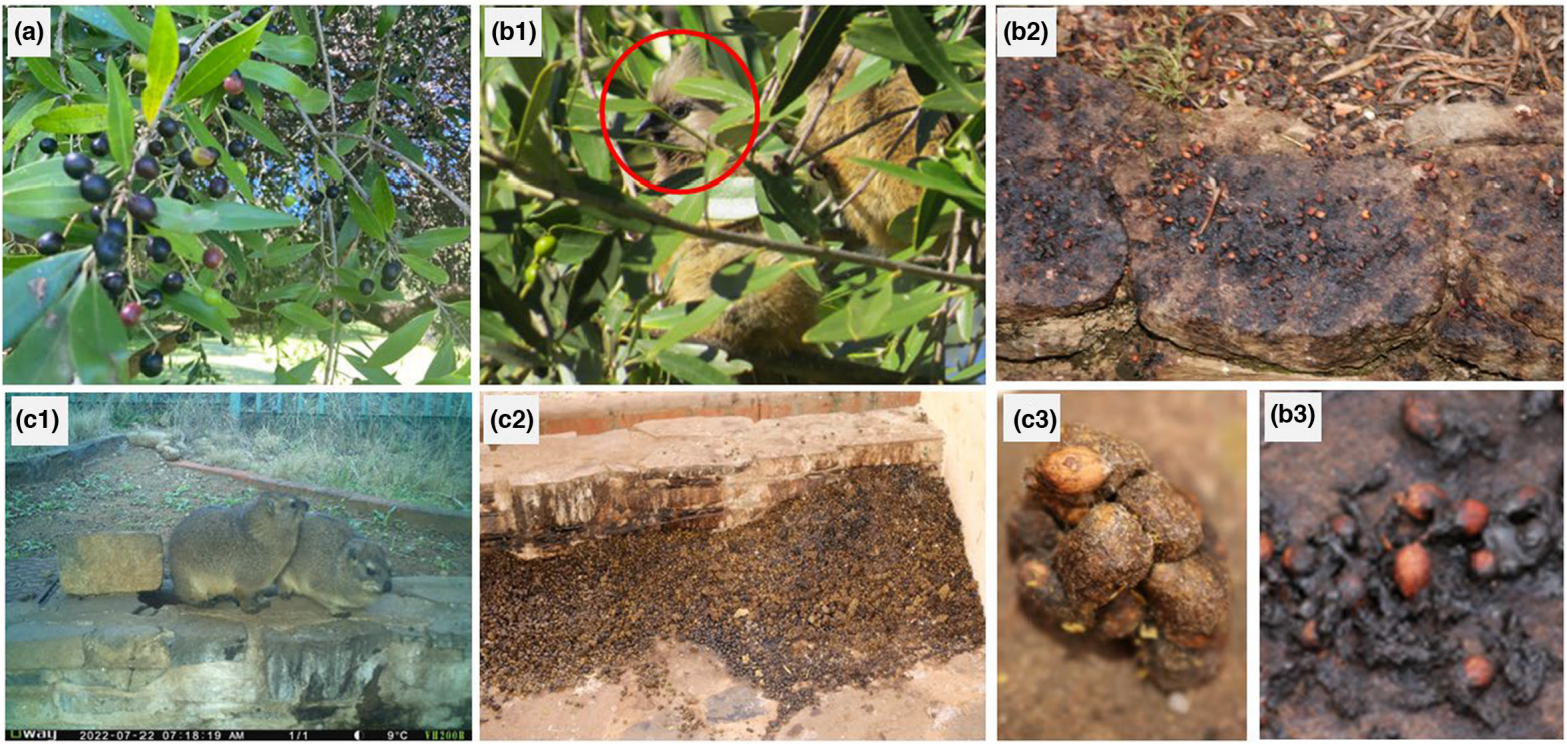
Defecated seeds that had passed through vertebrates' gut and trapped faecal samples were collected for seed germination trials during the study. A: ripe fruits of *O. subsp. africana*, B1: speckled mousebird *Colius striatus* (in red circle) foraging on *O. subsp. africana* fruits, B2‐3: bird‐defecated seeds in the roosting site; C1: Rock hyrax *Procavia capensis*; and C2‐3: defecated seeds traps from Rock hyrax (mammals) fecal sample.

*Olea europaea* subsp*. africana* is also an important taxon for the sustenance of the Gh7 Winburg Grassy Shrubland vegetation of the Dry Highveld Grassland bioregion (Mucina & Rutherford, 2006). According to Van Wyk and Van Wyk (2013), *O. europaea* subsp*. africana* grows in open woodlands, rocky mountain slopes, and along watercourses. Human manipulation of the land has significantly altered the natural vegetation in the grassland biome (Rutherford et al., 2012). For example, in the Hugumburda dry Afromontane forests of Ethiopia, Ourge et al. (2018) reported overexploitation of *O. europaea* subsp*. africana* due to human activities (farm implements, fuelwood, construction material, and medicine), and locally endangered the species. Furthermore, it was estimated that the total biomass of *O. europaea* subsp*. africana* wood harvested from 8103 ha was quantified to be between 2000 and 5000 tons per year (Ourge et al., 2018). In addition, the overuse of *O. europaea* subsp*. africana* material in South Africa in the production of commercial products and herbal medicines (Long et al., 2010; Msomi & Simelane, 2017), and sapwood for furniture, firewood, and fence posts (Pote et al., 2006) could threaten its conservation status in the near future. This species produces a small ovoid drupe fruit with spherical, thinly fleshy pulp which ripens purple‐black (March–July, Figure 2a; Vukeya et al., 2022), and seeds are indehiscent, stony, with hard endocarp (Figure 2c4; Cuneo et al., 2010; Vukeya et al., 2021, 2022).

### 2.3 Spatial data collection and image processing

In this study, the Classifier and Regression Trees (CART) algorithm was used to create a classifier model that classifies a region of interest based on the feature specified. The Google Earth Engine (GEE) platform was utilized to develop supervised classified images. Supervised classification is based on the selected sample pixels in an image that represent a specific class and then directs the image processing software to use these training sites as references for the classification of all other pixels in the image (Pech‐May et al., 2022). The Tier 1 USGS Landsat 8 surface reflectance dataset was employed and filtered to a region of interest “Free State Province—FSP.” Thereafter, filtered to the date range (“01 January 2020 to 31 December 2020”) and was sorted according to the cloud cover. To enhance land cover characteristics, we transformed the original Landsat image to Landsat bands’ true color composites for visual interpretation purpose.

A pixel‐based supervised classification with a maximum likelihood classifier was used to classify images. The land cover features were based on the observed biophysical cover on the earth's surface. We extracted representative samples (training points/polygons) of reflectance spectra for four landcover classes of interest: vegetation (woodland, grassland, sparse vegetation, and bare land), water body, human settlement‐mining, and agriculture and then merged to obtain a feature collection. The human settlement was merged with mining since they occupied small areas. This input imagery was further sampled using particular bands from the Landsat imagery to get a renewed feature collection of training data and the classifier model is trained using the CART Algorithm.

An accuracy assessment was further performed to determine the exactness of the proposed model and the results are plotted using a confusion matrix. By applying the CART algorithm for image classification, an accuracy of 80% was achieved.

The supervised classified image was exported to google drive and imported to Quantum Geographical Information System version 3.22 (QGIS). Thereafter, we overlay the spatial distribution of *O. europaea* subsp*. africana* in the Free State Province. The spatial distribution data were sourced from the SAPIA database 2022, National Vegetation Databases, and Global Biodiversity Information Facility (GBIF).

### 2.4 Seed collection and germination trials

Foraging organisms were documented using non‐invasive camera traps (Mokotjomela & Hoffmann, 2013; Vukeya et al., 2022). The two camera traps were set up in 12 different patches with stands of *O. europaea* subsp*. africana* to capture the foraging organisms. They were monitored after 24 h including day and night per month for 6 months of optimal fruiting periods: February—July 2021. We collected seeds that were trapped in the faecal samples of different animals including birds and mammals under the canopy of *O. europaea* subsp*. africana* during the fruiting period. Different faecal samples were dried at room temperature and seeds were extracted. Seed morphology was used to identify targeted seeds of *O. europaea* subsp*. africana*. In addition, more seeds were obtained by collecting faecal material from the roosting places.

The germination trials were carried out in the greenhouse of the Free State National Botanical Garden (FSNBG) between August 2021 and April 2022. The FSNBG has a Quonset‐style greenhouse of approximately 20 × 6 m in size and is equipped with a controlled irrigation system. Prior to germination trials, sowing trays were sterilized with bleach and water against the growth of fungi and insect pests (Nichols, 2005; Vukeya et al., 2021).

Three seed treatments were compared with the experimental control (intact fruit): de‐pulped seeds, seeds defecated by mammals (Rock hyrax *Procavia capensis*), and those defecated by frugivorous birds (Figure 2; Vukeya et al., 2021). The regular bird species were Olive thrush *Turdus olivaceus*, African red‐eyed bulbul *Pycnonotus nigricans*, Cape robin‐chat *Cossypha caffra,* Speckled pigeon *Columba guinea*, Cape white‐eye *Zosterops capensis*, and White‐backed mousebird *Colius colius* (10.9–252 g in size). All selected dispersal vectors had the “Least concern” IUCN conservation status, and birds were reportedly capable of long‐distance dispersal (Mokotjomela et al., 2013). For experimental control, 100 ripe fruits of *O. europaea* subsp*. africana* were also harvested from different trees. Since Meyer and Witmer (1998) and Traveset et al. (2007) have shown that fruit pulp contains germination inhibitors, other seeds were manually de‐pulped and then sown without any treatment. We considered the defecated seeds as those that had passed through the vertebrate's gut and had been found embedded in the faecal samples (Figure 2; Mokotjomela et al., 2021; Vukeya et al., 2021), and two types of defecated seeds were used. First, we used the seed defecated by the Rock hyrax since we did not have sufficient seeds from other mammals. The Rock hyrax is a small, tailless, rodent‐like animal with a long body of approximately 44 to 50 cm and weighing around 4 kg (Kingdon et al., 2013). Secondly, the seeds that had been ingested and treated by birds. Only seeds that were free from insects and pathogens were collected from roosting sites. Overall, 100 seeds were used per treatment. Each treatment comprised 10 seeds per tray and was replicated 10 times. Seeds were sown using a potting mixture; thereafter, germination trays were kept in the FSNBG greenhouse. The potting mixture contained Clivia mix with a well‐drained organic medium. Watering was done as necessary, either in the mornings before 10:00, or later, after 17:00. Temperatures were maintained at a level of ambient environmental conditions. The seed germination was monitored daily. The germination experiment was terminated when no new seedlings were recorded over a period of 6 weeks.

The seed germination speed was calculated according to the method by Ellis and Roberts (1980) and Mokotjomela et al. (2021) as a ratio of the number of seedlings that germinated to the total number of weeks from the sowing date to the duration of the experiment.

### 2.5 Estimating potential seed dispersal distances: Product gut retention time and animal daily movements (movement capacity)

To determine potential seed dispersal distances, we used allometric (i.e., as a function of animal body mass (BM)) mechanistic models to predict gut retention time (GRT) in hours for ingested seed, and movement capacity (MC) in Km for potential dispersal range for the two groups of organisms that influenced recruitment processes in *O. europaea* subsp*. africana*. For birds, the product of gut retention time and flight speed for birds and home range for mammals (i.e., movement capacity—MC) was considered to estimate a potential seed dispersal distance (Mokotjomela et al., 2013; Msweli, 2021; Schurr et al., 2009). We selected vertebrate species (mammals and birds) that were documented as consuming *O. europaea* subsp*. africana* fruits and have overlapping distributional records in the Free State Province.

For bird species, the relationships were estimated using equations from Robbins (1993) and Calder (1996) as follows:

(1) $${\mathrm{GRT}}_{\left(b\right)}=1.6\;{\left\{\mathrm{BM}\right\}}^{0:33\;\mathrm{kg}}$$

(1.6 and 0.33 are allometric constants), and

(2) $${\mathrm{MC}}_{\left(b\right)}=15.7\;{\left\{\mathrm{BM}\right\}}^{0:17\;\mathrm{kg}}$$

(15.7 and 0.17 are allometric constants; subscript “b” denotes birds).

Since the selected mammals were non‐ruminant species (hindgut fermenters), we estimated the GRT (in hours) using the allometric equation from Steuer et al. (2011) as follows:

(3) $${\mathrm{GRT}}_{\left(m\right)}=31.0\;{\left\{\mathrm{BM}\right\}}^{0.01}$$

(31.0 and 0.01 are allometric constants).

Although the MC is not consistent because of seasonality, age and sexual dimorphism, dietary type, body mass, and local availability of survival resources (McQualter et al., 2015; Msweli, 2021; Saïd & Servanty, 2005), the MC (in km) were estimated using appropriate equations derived and modified from du Toit (1990):

(4) $${\mathrm{MC}}_{\left(m\right)}=0.024\;{\left\{\mathrm{BM}\right\}}^{0.18}$$

(0.024 and 0.18 are allometric constants; subscript “m” denotes birds).

The final dispersal distances, therefore, were estimated as the product of GRT and MC for birds and mammals respectively.

### 2.6 Statistical analysis

#### 2.6.1 Seed germination trials

Seed germination count data were obtained from a balanced experimental design and were used to generate the proportions of the total number of seeds germinated per tray. The proportions of the numbers of seeds germinated in each treatment were arcsine‐transformed to reduce inequality of variance (Zar, 2010) and compared using the univariate analysis of variance at a significant level of *p* ≤ .05 using Statistical Package for Social Science (SPSS, version 28). The counts of germinated seeds were treated as a response variable and different treatments were specified as the predictor variables. Dunnett post hoc test was used to compare all treatments against the experimental control (Zar, 2010).

Since the daily seeds' germination data were zero‐inflated (Zar, 2010), the weekly counts of geminated seeds in different treatments were used to estimate the germination speed as a total number of seeds germinated per tray derived by the number of weeks (after, Mokotjomela et al., 2021). A multiple comparison test using the Kruskal–Wallis Analysis of Variance was applied to compare seed germination speed in different treatments. The cumulative weekly germination data were also plotted against experimental time to determine the seed germination speed in each treatment.

## 3 RESULTS

### 3.1 Land cover use and species distribution

**FIGURE 3:**
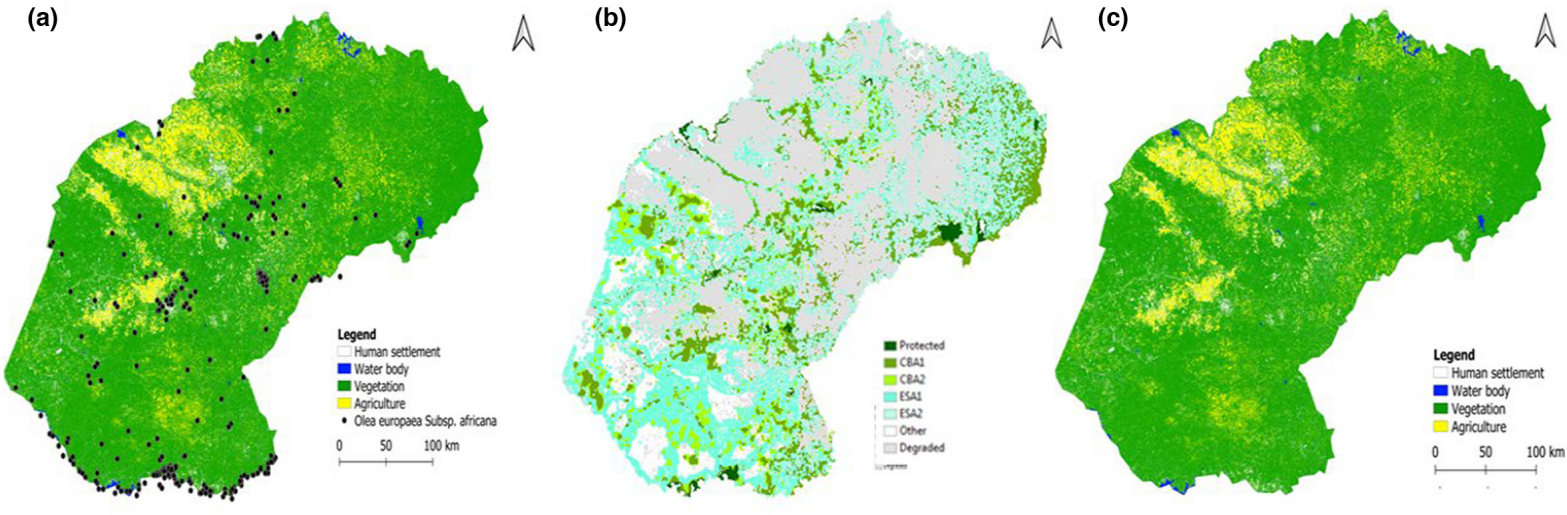
Spatial analyses of the study area: (a and c)—land cover classification overlayed with geographical distribution of *O*. subsp. *africana* within Free State Province (black dots); and (b)—map of Critical Biodiversity Area's (CBAs) and Ecological Support Area's (ESAs) within Free State Province (Biodiversity plan: Collins & Free State Department of Economic, 2016).

With reference to the Critical Biodiversity Area's map of Free State Province (Collins & Free State Department of Economic, 2016; Figure 3b), overall, the results showed that 39% of the natural habitat has been transformed by human‐mediated activities. Agricultural activities accounted for 27% while mining activities and human settlement accounted for 12% of natural habitat loss (Figure 3c). The results show that habitat fragmentation isolates the *O. europaea* subsp. *africana* populations largely from the north tip to the central and east of the Free State Province (Figure 3a).

### 3.2 Seed germination trials

**FIGURE 4:**
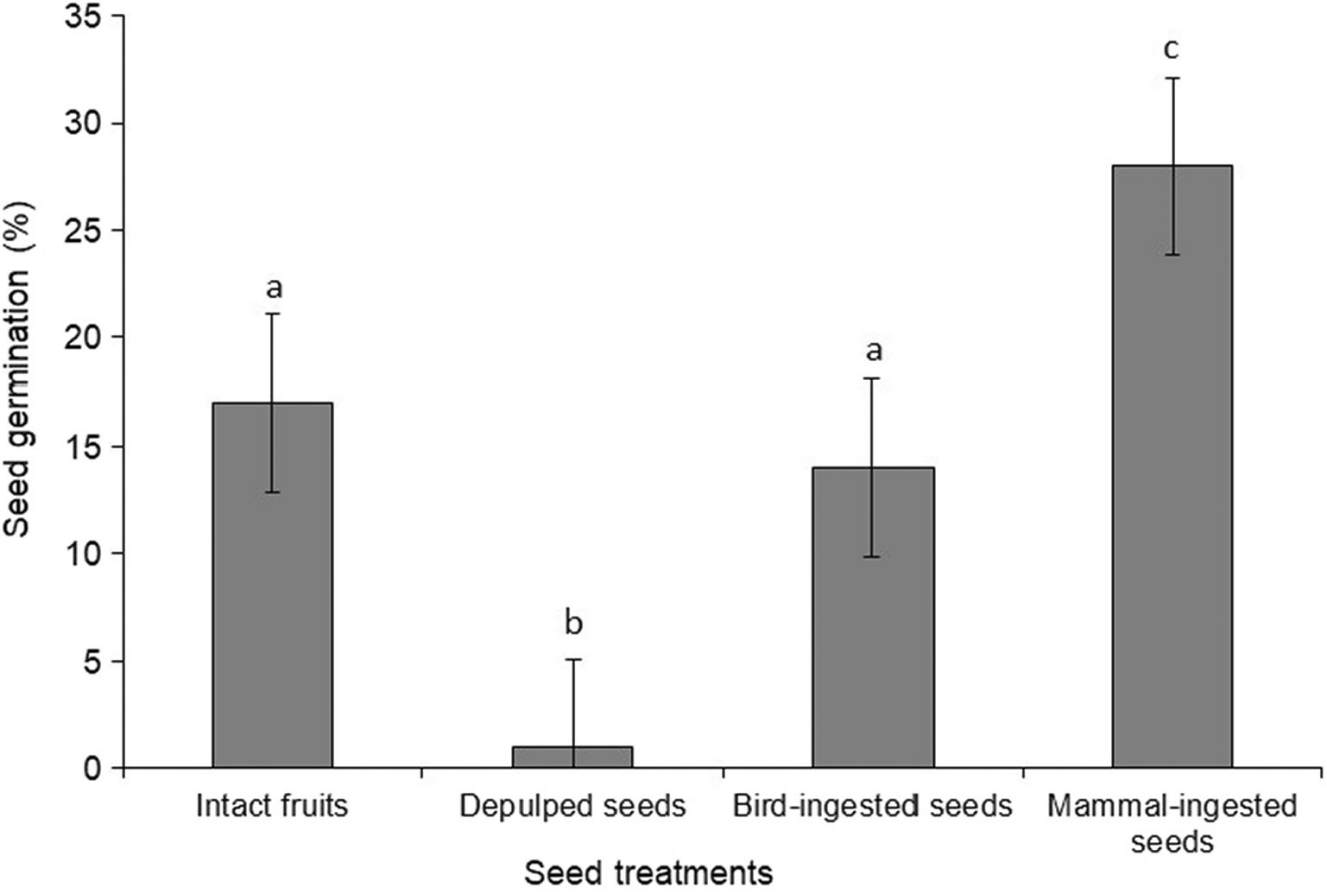
Mean % number of *O*. subsp. *africana* seeds germinated across four treatments: Intact fruits, de‐pulped seeds, bird‐ingested seeds, and mammal‐ingested seeds. Bars represent the standard error of the mean, and different letters denote significant differences between seed treatments.

Overall, the comparisons of the seed germination between the treatments and experimental control were highly significant (*F*_(3, 1316)_ = 7.6; *p* < .0001; Figure 4). Dunnett post hoc test showed that there was a significantly higher number of seeds germinated in treatment by mammal ingestion compared with other seed treatments and the experimental control (intact fruits). In support of the study prediction, mammal‐ingested seeds had the highest seed germination (mean ± SE: 28.0 ± 4.12%; *N* = 330; Figure 4).

Also, there was no statistically significant difference between seed germination of the bird‐ingested seeds and intact fruits as the experimental control (14.0 ± 2.67%; 17.0 ± 5.59%, respectively), although Dunnett post hoc test showed that both were significantly greater than the de‐pulped seeds (1.0 ± 1.0%; Figure 4).

**FIGURE 5:**
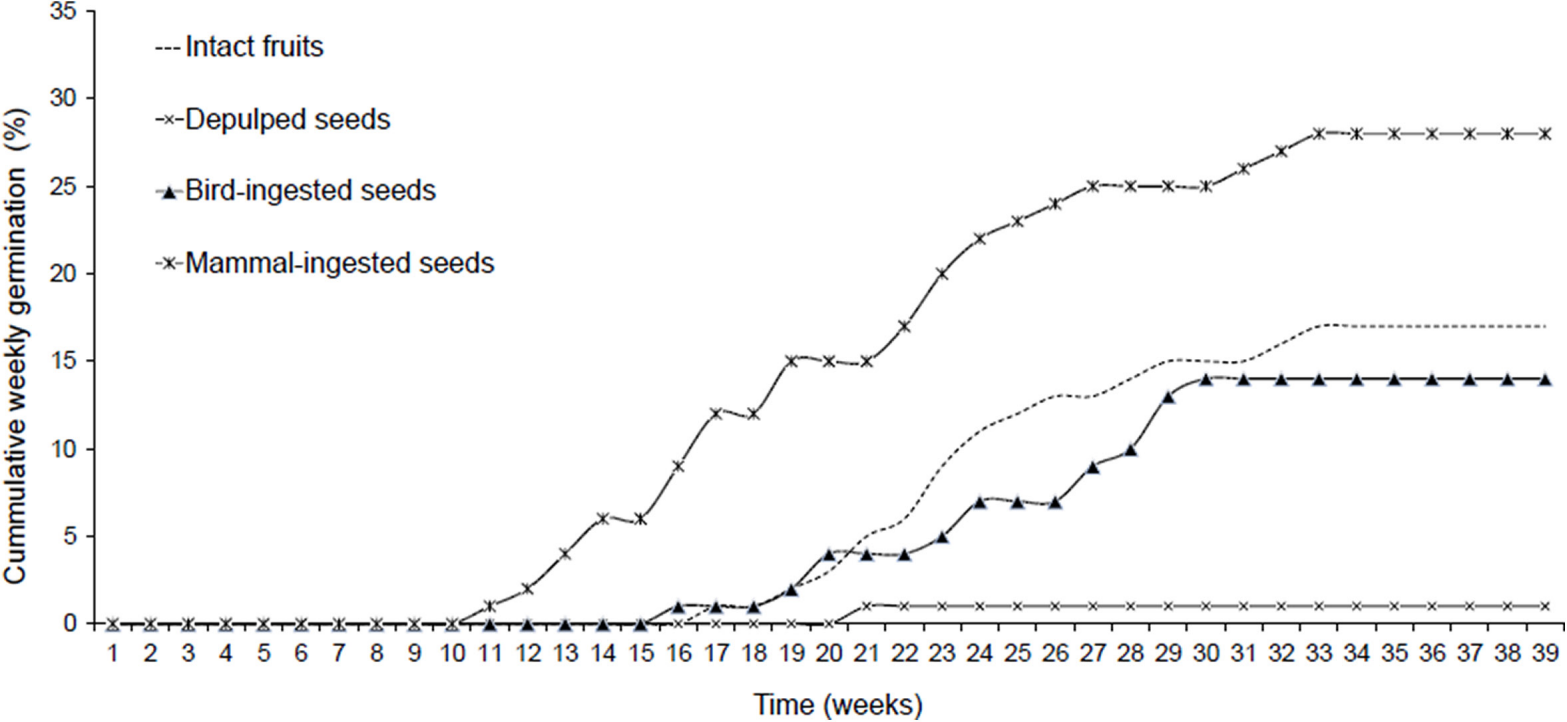
Cumulative weekly seed germination (%) between four seed treatments: Mammal‐ingested seeds, bird‐ingested seeds, de‐pulped seeds extracted from the fruit, and Intact fruit as unprocessed fruit for *O*. subsp. *africana*.

There was no seed germinated within the first 10 weeks after the seeds were sown. However, *Olea europaea* subsp*. africana* had the seed germination speed significantly improved after passage through the mammal gut (1.49 per week) as opposed to other seed treatments, with the first seedlings emerging in week 11 (*H*_(3, *N* = 132)_ = 18.0; *p* = .0002; Figure 5).

## 4 POTENTIAL SEED DISPERSAL DISTANCES

Birds had relatively large potential seed dispersal distances ranging from 9.4 km to 53 km as opposed to mammals (1.5 km–4.5 km; Table 1), and these results conformed with the reported 1 km threshold for long‐distance dispersal. While birds were partly migratory and resident in behavior, all mammals foraged at a central place, complemented by birds that disperse seeds over relatively longer distances in this study (Table 1).

**TABLE 1:** Potential seed dispersal effectiveness for the two groups of organisms that influenced recruitment processes in *O.* subsp*. africana* population: Potential dispersal distance, gut retention time and foraging behavior.

Species	Family	Body size (kg)	Gut retention time (GRT) in hours	Movement capacity (km/hour)	Potential dispersal distance (km)	Foraging behavior
	Birds—frugivores					
*Turdus olivaceus*	Turdidae	0.08	0.69	10.18	25.3	Resident and migrant (Hockey et al., 2005)
*Pycnonotus nigricans*	Pycnonotidae	0.03	0.51	8.69	15.9	Resident and nomad (Hockey et al., 2005)
*Colius colius*	Coliidae	0.04	0.55	9.08	18.1	Migratory and resident (Hockey et al., 2005)
*Zosterops capensis*	Zosteropidae	0.01	0.36	7.28	9.4	Resident (Hockey et al., 2005)
*Columba guinea*	Columbidae	0.34	1.13	13.10	53.0	Resident
*Cossypha caffra*	Muscicapidae	0.03	0.49	8.57	15.2	Resident (Hockey et al., 2005)
	Mammals—frugivores					
*Procavia capensis*	Procaviidae	4.00	31.43	0.03	3.5	Territorial and central place foraging
*Hystrix africae australis*	Hystricidae	15.00	31.85	0.04	4.5	Territorial and central foraging
*Rhabdomys pumilio*	Muridae	0.04	30.02	0.01	1.5	Central place foraging
*Mastomys natalensis*	Muridae	0.04	30.03	0.01	1.5	Central place foraging
*Micaelamys namaquensis*	Muridae	0.05	30.10	0.01	1.5	Central place foraging
*Chlorocebus pygerythrus*	Cercopithecidae	5.50	31.53	0.03	3.7	Nomadic and multiple central place foraging (Chapman et al., 1989)
*Papio ursinus*	Cercopithecidae	14.00	31.83	0.04	4.4	Multiple central place foraging and Nomadic (Pamla, 2016)
*Geosciurus inauris*	Sciuridae	0.65	30.87	0.02	2.5	Central place foraging

## 5 DISCUSSION

The habitat fragmentation mediated by human activities has drawn the attention of the world to the conservation of threatened biodiversity as an example of human–wildlife conflict. In this study, we present evidence of the potential negative impacts of habitat fragmentation on the distributional range of the keystone *O. europaea* subsp. *africana* population focusing on the Free State Province, South Africa**.** We infer that the continuous habitat fragmentation may have substantially reduced the population range of *O. europaea* subsp. *africana* population which could lead to the local extinction of *O. europaea* subsp. *africana* and other species, directly dependent on its ecological goods and services in the grassland biome. We also use the plant–animal interactions model (i.e., birds and mammals foraging on fruits of *O. europaea* subsp. *africana*) to highlight the critical role of vertebrate seed dispersal in plant recruitment, and in offsetting the negative impacts of habitat fragmentation on *O. europaea* subsp. *africana*.

Our finding that 39% of the natural habitat has been transformed confirmed previous reports (e.g., Carbutt & Kirkman, 2022; Matsika, 2008; O'connor & Kuyler, 2009; Skowno et al., 2019) showing that agricultural production, human settlement, and mining activities have reduced the space available for *O. europaea* subsp. *africana* population in the Grassland Biome. Specifically, Skowno et al. (2019) reported a significant habitat loss of grasslands that occurred prior to the 1990s and partial loss between 1990 and 2014 from the expansion of agricultural crop production in the northern part of the Free State Province. Above all, the Grassland Biome in South Africa has been a low national priority for conservation with only 3% being formally protected (Neke & du Plessis, 2004; SANBI, 2013; Skowno et al., 2019). Matsika (2008) also identified the establishment of alien forest plantations, and human settlement as other major drivers of habitat cover loss in the grassland biome. Indeed, the socio‐economic and infrastructural development pressures associated with the needs of the rapidly growing human population could also exacerbate the loss of the Grassland Biome (Goble et al., 2014; Greyling, 2012; Kanianska, 2016; Laurance et al., 2014). We suggest that since grasslands constitute an important global carbon storage (Yuan et al., 2021), there is urgent need for mitigating fragmentation as part of climate change adaptation.

We found that the seeds of *O. europaea* subsp*. africana* germinated at a slower rate (up to 33 weeks), possibly because of the high resistance of their seed coat to scarification, within the gut of the vertebrates, to early release of seed dormancy (Mokotjomela, 2012; Vukeya et al., 2021, 2022). Slow germination might retard recruitment since Schupp (1993) argued that of utmost importance in the effectiveness of seed dispersal is their ability to germinate in the new environment. Indeed, where habitat fragmentation is rapid and continuous, the remaining species may survive through their ability to disperse and effectively colonize new microsites for the establishment of new self‐sustainable populations (Howe & Smallwood, 1982; Traveset, 1998; Wang & Smith, 2002). Our results are not different since Bekele (2000) demonstrated that *O. europaea* subsp. *africana* seeds may persist for up to 35 months of seed dormancy, and this could be an adaptational mechanism to allow seed maturation and reduce seasonal competition for germination resources with other plant species (Orrock & Christopher, 2010; Vukeya et al., 2021). Alternatively, this delayed germination may provide a competitive advantage for resources (light and nutrients; Donohue et al., 2010; Dubois & Cheptou, 2012) and avoiding exposure to predators and pathogens (Parsons, 2012; Traveset & Verdú, 2002). However, seeds of *O. europaea* subsp. *africana* had significantly increased germination after passing through the gut of mammals (non‐ruminants), suggesting that the seeds benefited from scarification of the coat in the digestive tract. With such species displaying keystone functions in South Africa, we propose an elevated conservation attention through arresting the human‐mediated habitat fragmentation.

Additionally, increased germination could be a likely result of longer gut retention times of seeds by the relatively large‐bodied mammals compared to birds (Herrera, 1989; Mokotjomela et al., 2016; Schurr et al., 2009), which dominant dispersal vectors in the study site (Vukeya et al., 2022). Long gut‐retention times of seeds may allow the digestive fluids in large mammals to partially corrode the seed coat (Jordano, 2000; Stevens and Hume, 1998; Traveset et al., 2007). Such treatment increases its permeability for imbibition of water as an essential resource for germination (Mokotjomela et al., 2015, 2021). It has been reported the cracking effect of teeth on seed coats during the grinding action of food to improve their germination (Lowry, 1996; Samuels and Levey, 2005). This finding points out to the need for integrated conservation of mutualists instead of individual species as way of protecting the whole habitat (New, 2017).

Conversely, we found non‐significant differences between the germination of bird‐ingested seeds and the experimental control (intact fruit), partially because of the short gut‐retention time in combination with high resistance of the seed coat to releasing seed dormancy (Mokotjomela et al., 2015). In the same manner, this study indicates unchanged *O. europaea* subsp. *africana* seed germination after passing through bird guts has been previously reported by Vukeya et al. (2021). It is a likely result of possible frugivorous bird gut adaptations to the egestion of indigestible fruit material such as seeds (Jordano, 2000; Mokotjomela et al., 2021). Although birds did not increase seed germination of *O. europaea subsp. africana*, they have been reported to provide important seed dispersal over long distances (Chama et al., 2013; Mokotjomela et al., 2016). Fruit pulp sugar content results in osmotic pressure decrease and even precludes seed germination (Cipollini and Levey, 1997; Evenari, 1949; Traveset et al., 2007). However, we found that the intact fruit as experimental control germinated significantly better than manually de‐pulped seeds and thus speculate that the pulp content of the fruit certainly modifies the seed environment conditions to release and then re‐adjust the dormancy within the seed. Poor germination of de‐pulped seeds observed in this study may be attributed to the stony seed coat that slows down the uptake of moisture and oxygen to seed embryos (Bekele, 2000; Negash, 2010). Whereas the retarded seed germination could be seen as a recruitment compromise, Vukeya et al. (2021) reported that seeds are capable of sensing conducive conditions for survival, and thus, further research is required to elucidate on observed low germination.

Birds had a high potential for dispersing seeds over a longer distance compared to mammals (9.4 km to 53 km vs 0.3 km–2.5 km) since they can fly between isolated *O. europaea* subsp*. africana* populations partly searching for fruits (Lenz et al., 2011; Mokotjomela et al., 2013; Tellería et al., 2008). Studies in the eastern part of South Africa have shown that birds fly between forest patches/fragments in search of food and mates, and thus mediated important dispersal services (Saracco et al., 2004; Tellería et al., 2008). Similarly, we propose that the study species could complementarily mediate seed dispersal across the fragments in the study area since they are ubiquitous (IUCN, 2023). However, the efficiency of mammal seed dispersal to assist the natural restoration of degraded *O. europaea* subsp*. africana* population may be limited by the central place foraging behavior (Chapman et al., 1989). For example, the clumped deposition of seeds may result in increased seedling competition in natural conditions (Vukeya et al., 2021), which may retard recruitment due to density‐dependent mortality, especially in the fragmented habitats (Augspurger & Kelly, 1984; Schwinning & Kelly, 2013). Nevertheless, other reports have shown that secondary dispersal vectors such as baboons and livestock may target fecal material rich in seeds during times of food scarcity (Mokotjomela, 2012), and this may offset limited dispersal distances reported for the mammals such as rock hyrax in this study. In the tropical rainforest of Mexico, Urrea‐Galeano et al. (2019) reported that Dung beetle activity extended the seed's dispersal by aggregation of seeds after being deposited by mammals in fecal clumps, although not increasing the germination rate.

## 6 CONCLUSIONS

We have shown that the distribution range of *O. europaea* subsp. *africana* population is largely driven by habitat fragmentation through agriculture production, human settlement, and mining activities in the Free State Province. Our findings are consistent with the current reports of natural habitat protection in South Africa (Skowno et al., 2019). We also showed that plant–animal interaction between *O. europaea* subsp. *africana* and vertebrate frugivores (birds and mammals) are critical to restoring degraded woody species because the vertebrates partly improved seed germination (Mokotjomela et al., 2021; Vukeya et al., 2021, 2022), and dispersed seeds to new microsites, even if with no improvement of seed germination was observed. The long‐distance seed dispersal mediated by birds as a proxy of quality for dispersal effectiveness (sensu, Schupp et al., 2010), will be dependable for the recruitment of *O. europaea* subsp. *africana* in South Africa. In fragmented habitats, birds may fly between different patches in search of food resources (Gumede et al., 2022; Maseko et al., 2020). We argue that the shorter dispersal distances by mammals could undermine the role of extended gut retention times of seeds owing to their big body sizes. However, we cannot rule out the possibility of longer dispersal distances than estimated in this study because of stochastic ecological events such as wildfires and floods (Mokotjomela et al., 2013; Sarraco et al., 2004; Tellería et al., 2008). New research must focus on secondary dispersal vectors of the clumped dispersal observed for some mammals as they may have a disproportionate contribution to plant recruitment (Hämäläinen et al., 2017), and a rigorous evaluation of the demographics of the target species remains to be done at the study site. Finally, we recommend that restoration/conservation efforts could benefit from the collection and storage of mammal‐ingested seeds for seedling production.

## AUTHOR CONTRIBUTIONS

**Loyd Rodney Vukeya:** Conceptualization (equal); data curation (equal); formal analysis (equal); investigation (equal); methodology (equal); writing – original draft (equal). **Thabiso Michael Mokotjomela:** Conceptualization (lead); data curation (lead); formal analysis (equal); methodology (lead); software (lead); supervision (lead); writing – original draft (equal); writing – review and editing (equal). **Leslie Powrie:** Data curation (equal); methodology (equal); visualization (equal); writing – review and editing (equal). **Lufuno Nenungwi:** Data curation (equal); investigation (equal); resources (equal).

## CONFLICT OF INTEREST STATEMENT

The authors declare that no conflict of interest exists.

## Data Availability

Mokotjomela, Thabiso (2022), distribution of *Olea europaea subspecies africana* in Free State South Africa, Dryad, Dataset, https://doi.org/10.5061/dryad.2280gb5w8.
